# Monoclonal Antibodies in Multiple Sclerosis: Present and Future

**DOI:** 10.3390/biomedicines7010020

**Published:** 2019-03-14

**Authors:** Natalia V. Voge, Enrique Alvarez

**Affiliations:** Rocky Mountain Multiple Sclerosis Center at the University of Colorado, Department of Neurology, University of Colorado School of Medicine, Academic Office 1, Mail Stop B-185, 12631 East 17th Avenue, Aurora, CO 80045, USA; natalia.voge@ucdenver.edu

**Keywords:** monoclonal antibodies, anti-CD20, Ocrevus, Rituxan, Tysabri, multiple sclerosis, clinical trial, disease modifying therapy

## Abstract

The global incidence of multiple sclerosis (MS) appears to be increasing. Although it may not be associated with a high mortality rate, this disease has a high morbidity rate which affects the quality of life of patients and reduces their ability to do their activities of daily living. Thankfully, the development of novel disease modifying therapies continues to increase. Monoclonal antibodies (MABs) have become a mainstay of MS treatment and they are likely to continue to be developed for the treatment of this disease. Specifically, MABs have proven to be some of the most efficacious treatments at reducing relapses and the inflammation in MS patients, including the first treatment for primary progressive MS and are being explored as reparative/remyelinating agents as well. These relatively new treatments will be reviewed here to help evaluate their efficacy, adverse events, immunogenicity, and benefit-risk ratios in the treatment of the diverse spectrum of MS. The focus will be on MABs that are currently approved or may be approved in the near future.

## 1. Introduction

Monoclonal antibodies (MABs) are one of the preferred treatments for multiple sclerosis (MS) due to their target specificity and usually high efficacy [1]. These have usually targeted the immune system, which plays a key role in the pathogenesis of MS, especially during the early inflammatory stages. MABs bind very specifically to epitopes or parts of larger proteins (antigens) that allow them to mediate their effects on very specific pathways. In MS, MABs are able to more specifically neutralize key immune players that negatively impact the central nervous system. Because of their specificity, they tend to have few off target effects and less drug–drug interactions minimizing their side effects, which tend to arise from their downstream effects on the immune system and reactions to the drugs themselves, although this problem has been greatly reduced with the advent of humanized MABs [1,2].

**Figure 1:**
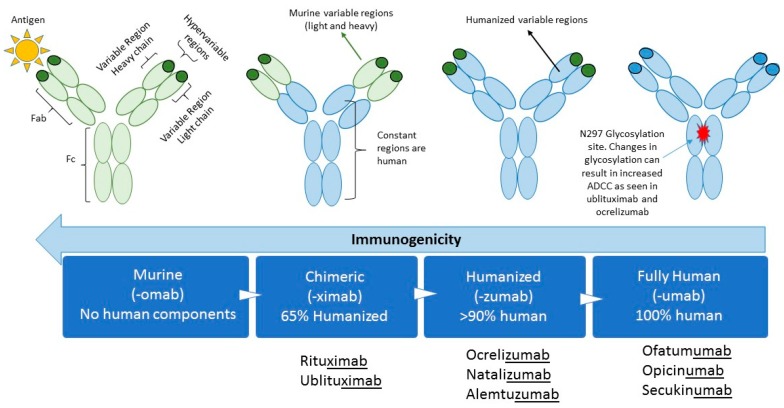
Bioengineering techniques have resulted in progressively more humanized antibodies. The figure shows the different fractions of the monoclonal antibodies and whether they represent mouse (green) or human (blue) sequences. As the sequences become more humanized their immunogenicity decreases. Glycosylation occurs at amino acid N297 and affects ADCC. Fragment antigen binding (Fab), Fragment crystallizable (Fc), Antigen-dependent cellular cytotoxicity (ADCC).

Therapeutic MABs were originally developed from non-human species such as mice. The first MAB to be approved was muromonab-CD3 (Orthoclone OKT3) which targeted the CD3 surface protein of T lymphocytes to help prevent organ rejection in 1985 [3]. Reactions to murine MABs were soon associated with the development of antidrug antibodies (ADAs) against the murine-based protein with repeated exposures [4]. In order to reduce the potential immunogenicity of murine MABs, chimeric mouse-human antibodies were developed (Figure 1). This occurs by inserting the antigen-specific variable domain of a mouse antibody on the constant domains of a human antibody, producing antibodies that are up to 65% humanized [5,6]. Humanized antibodies that contain human light and heavy chains but retaining murine hypervariable regions being greater than 90% humanized will be developed. Transgenic mouse strains, which express human variable domains, have allowed the production of MABs with a reduced immunogenic potential, making them “fully humanized” and with properties similar to any human antibody [7]. These antibodies can be further modified to change their properties, for example by altering the glycosylation at amino acid N297 to increase their antigen-dependent cellular cytotoxicity (ADCC).

Natalizumab was the first MAB to be approved by the United States Food and Drug Administration (FDA) for the treatment of MS in 2004 [8], heralding the age of MABs for the treatment of MS. Other MABs have followed including alemtuzumab and ocrelizumab. Rituximab and ofatumumab have been used off label in the treatment of MS and ublituximab and ofatumumab are undergoing Phase III studies for approval in MS.

Opicinumab targets leucine-rich repeat and immunoglobulin domain-containing neurite outgrowth inhibitor receptor-interacting protein 1 (LINGO-1) [9], the remyelinating/reparative MAB that has been the most studied in MS. It has undergone phase II trials with mixed results and is currently being evaluated in another phase II study (AFFINITY). The MABs previously listed will be the focus of this review and other treatments completing phase II studies in MS can be found in Table 1; including some preliminary compounds in phase II such as Elezanumab, VAY736, and the HERV-W Env Antagonist GNbAC1. 

In the past decade, several MABs were developed in an attempt to find a more efficacious and safe therapeutic option for MS patients; rigorous clinical trials have demonstrated lack of efficacy of as well as serious adverse events from some of them. For example, daclizumab (Zinbryta) [10] binds CD25, a subunit of the IL-2 receptor, was approved in 2016 for the treatment of MS, but was voluntarily withdrawn from the market due to reports of encephalitis in March 2018. Additionally, MABs that have been withdrawn or discontinued from further testing are listed in Table 2. 

**Figure 2:**
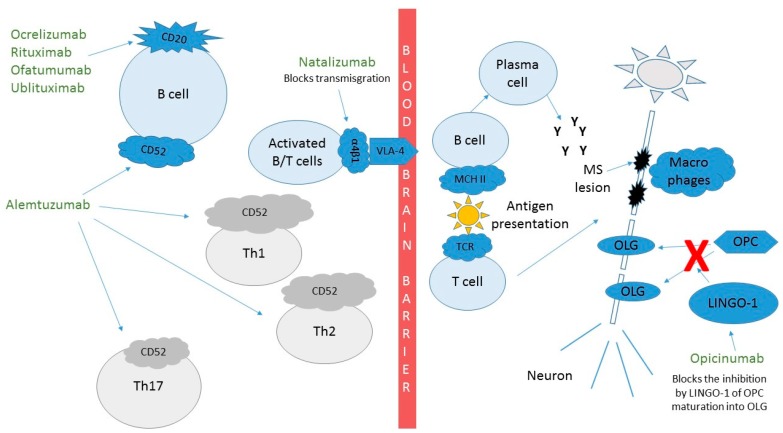
Mechanism of action of monoclonal antibodies in the treatment of multiple sclerosis. Ocrelizumab, rituximab, ofatumumab and ublituximab target CD20 expressing cells. Natalizumab targets transmigration of lymphocytes through the blood brain barrier. Alemtuzumab targets CD52 expressing cells. Opicinumab helps OPC differentiate into myelin producing OLGs. Cluster of differentiation (CD), T helper cells (Th), Leucine rich repeat and Immunoglobin-like domain-containing protein 1 (LINGO-1), oligodendrocyte (OLG), oligodendrocyte precursor cell (OPC).

Although the MABs have some of the best proven efficacy in the treatment of MS, side effects remain a concern for some providers and patients alike. Figure 2 shows how these MABs affect the pathophysiology of MS and their efficacy is described below for each MAB. The incomplete understanding of the immune system and how alterations to it can lead to infections, other autoimmune conditions, and possibly neoplasms is something that we will learn with experience, especially as we look to prescribe these treatments to our patients for many years. Additionally, the relatively unpredictable events secondary to MABs leading to immune reactions, including serum sickness and anaphylaxis, can be reduced by humanizing MABs as described above. Infusion-related reactions (IRRs) can be common and are more likely related to cytokine release syndrome (CRS) than to reactions to the MABs themselves. CRS can be a serious adverse event but is more common in conditions where the target lymphocytes are in high abundance. For example, CRS is much more common and severe in the treatment of B-cell lymphomas than in the treatment of MS with anti-CD20 MABs. CRS manifestations include a wide clinical spectrum, which can vary from mild flu-like symptoms, to life-threatening manifestations including progression to uncontrolled systemic inflammatory response, vascular leakage, disseminated intravascular coagulation and multi-organ system failure [21,22]. Although CRS or milder versions of it have the highest incidence during the first infusion, they can occur with later infusions as well. These IRRs tend to occur during or soon after the infusions. Even some of the fully humanized MABs can be immunogenic, causing anaphylaxis or serum sickness in later infusions [23]. Pretreatment with steroids, acetaminophen, and/or an antihistaminic prior to infusion can reduce IRRs and CRS and are often used prior to medications that cause lymphocyte destruction.

Clinical evidence of continued disease activity, side effects, ease of administration, and costs are some of the aspects to consider when switching a patient to/from MAB therapies. Understanding the mechanism of action, how to monitor for adverse events and developing strategies for evaluating treatment failure are important tenants of personalized medicine and help to provide the safest treatment for patients with MS.

### 1.1. Natalizumab (Tysabri™)

Natalizumab was the first MAB approved by the FDA in 2004 for the treatment of MS (Table 1). Although it was temporarily withdrawn from the market due to cases of progressive multifocal leukoencephalopathy (PML), it was returned to the US market in 2006 after development of a risk evaluation and mitigation strategy (REMS) program to monitor the cases of PML [24] It is a recombinant humanized MAB to integrin α-4, which forms a heterodimer with β1. By binding the α4β1 integrin on the surface of activated inflammatory lymphocytes and monocytes, natalizumab blocks the interaction with VCAM-1 on endothelial cells preventing these inflammatory cells’ entry into the central nervous system (Figure 2). 

Natalizumab has shown great efficacy in the treatment of patients with MS. In the AFFIRM study, natalizumab reduced the annualized relapse rate (ARR) by 68% compared with placebo at one year, and with a significant reduction of 42% in the cumulative probability of sustained 3 months disability progression over two years [25]. Additionally, there was an 83% reduction in new or enlarging T2 lesions with a 92% reduction in contrast-enhancing lesions in the natalizumab group as compared to placebo. 

Natalizumab is well tolerated at therapeutic doses with a very low rate of infusion reactions. It has been documented that anti-natalizumab neutralizing antibodies decrease free and cell-bound drug levels [26]. These develop and persist for at least 42 days after treatment in approximately 6% of natalizumab-treated patients [25,27]. Persistence of anti-natalizumab antibodies has been associated with reduced efficacy and increased incidence of IRRs [25,27]. 

The most serious adverse event consist of the brain infection PML caused by the John Cunningham virus (JCV). The risk of PML has been associated with prior chemotherapy and immunosuppressant use as well as amount of time on the drug. Additionally, a positive stratify JCV titer has been associated with higher risk of PML and this increases with higher titers. The index to assess PML risk consist in measurement of antibodies to JCV. Patients with a positive index have a higher risk of PML, which increases particularly in patients with an index >1.5 [24]. 

A routine monitoring strategy of the JCV titer should be part of the drug monitoring strategy for natalizumab (usually every 6 months) along with routine basic laboratories including a comprehensive metabolic panel and complete cell count with differential. In JCV seronegative patients, the benefits of natalizumab treatment still greatly outweigh the potential risks of PML [28,29] Clinical and neuroimaging follow-ups are recommended for disease activity monitoring and detection of side effects. 

Other documented adverse events (AEs) with natalizumab include infections with herpes virus [27,30] and is contraindicated in patients that are severely immunocompromised. Melanoma risk may be higher in at risk-patients [31]. Liver injury can occur in patients with no previous history of liver disease, although rarely [32]. Hypereosinophilia may occur during treatment with this MAB [33]. 

### 1.2. Ocrelizumab (Ocrevus™)

Ocrelizumab is a cytotoxic recombinant humanized MAB that targets CD20 on circulating B-lymphocytes (Table 1, Figure 2). Ocrelizumab was the first anti-CD20 treatment approved for use in MS patients, although rituximab had been used off-label previously. In comparison to rituximab, ocrelizumab has a modified Fc region with enhanced ADCC [34]. As a humanized molecule, ocrelizumab is expected to be less immunogenic and might thus have fewer IRRs than rituximab [35]. Ocrelizumab reduces CD20 counts after infusion with a median time for B-cell counts to return to baseline of 72 weeks [36]. CD19 counts are often used instead of CD20 cell counts for monitoring these medications since both proteins are expressed in nearly the same cell populations and due to concerns that CD20 could be masked by a bound drug not allowing detection of cells but that did not lyse [36]. 

In relapsing MS, ocrelizumab showed efficacy superiority over interferon Beta-1a (IFN-β1a). The ARR was significantly reduced by 46% and 47% in the two OPERA studies [37]. Additionally, contrast enhancing lesions decreased by 94% and 95% and new or newly enlarging T2 lesions decreased by 77% and 83%. Confirmed disability progression at 12 weeks decreased by 40% in a pooled analysis of both studies.

In primary progressive MS, ocrelizumab reduced the confirmed disability progression at 12 weeks by 24% over placebo. T2-weighted lesion volume decreased by 3.4% in ocrelizumab-treated patients but increased 7.4% with a placebo. The brain volume loss in ocrelizumab-treated patients was 0.90% versus 1.09% with placebo (*p* = 0.02) [34]. 

Common adverse events include IRRs, which is the reason that premedication is recommended with methylprednisolone, acetaminophen and diphenhydramine. Although common, these are rarely serious. Additionally, nasopharyngitis (14.8%), upper respiratory tract infections (15.2%) [37], headache, and urinary tract infections have been seen in patients treated with ocrelizumab. An increased risk of neoplasms, particularly breast cancer were noted and will need to be studied more to understand if this was indeed related to ocrelizumab. It is recommended that vaccinations be administered at least six weeks prior to starting ocrelizumab, with avoidance of live or live attenuated vaccines during treatment [38,39]. Ocrelizumab is contraindicated in patients with active hepatitis B infections, thus patients should be screened prior to starting treatment. PML has been reported when transitioning from natalizumab or fingolimod, but should be regularly monitored for in patients on ocrelizumab long term [40]. Additionally, patients should have standardized monitoring during treatment with ocrelizumab, including immunoglobulin G levels, as these levels can decrease placing patients at increased risk of infection if their levels drop to very low levels [38]. 

### 1.3. Rituximab (Rituxan™)

Rituximab is a chimeric MAB that binds to CD20 and lyses B cells via complement-dependent cytotoxicity (CDC) and ADCC (Table 1) [1,41,42]. It achieves a >95% depletion of B cells, which is sustained at week 24. By 48 weeks, B cells remain at 30.7% of baseline [43]. After the initial infusion, a depletion of T cells is observed in CSF in addition to the expected decrease in B cells [41,44,45]. 

Rituximab, commonly prescribed off-label, is very effective in relapsing MS [43]. In the HERMES phase II study, patients in the rituximab group had a significant reduction in total number of contrast enhancing lesions over 24 weeks versus placebo (mean number 0.5 versus 5.5; relative reduction 91%). The proportion of patients in the rituximab group with relapses was decreased at week 24 (14.5% vs. 34.3%, *p* = 0.02) and week 48 (20.3% vs. 40.0%, *p* = 0.04) [43]. The OLYMPUS study in primary progressive MS failed to show a reduction in the confirmed progression of disability at 12 weeks, but did find a significant reduction of 48% in those aged <51 and of 59% in those with enhancing lesions at baseline [46]. 

Rituximab caused more IRRs within 24 h after the first infusion versus placebo [43]. Adverse reactions include serum sickness, PML, neutropenic fever, sinusitis, nasopharyngitis, upper respiratory infection, urinary tract infection, reactivation of hepatitis B virus, cardiac arrhythmias, cytopenias and malignancies, which have been associated with chronic B-cell depletion, among other less frequently reported [41,47]. Serious AEs were predominantly reported in patients >55 years of age [47]. The development of anti-chimeric neutralizing antibodies secondary to treatment with rituximab is reported in 26% of patients treated in progressive MS and in 37% in RRMS patients, which is partially the reason for the development of less immunogenic humanized MABs [48]. Recommended patient monitoring is similar to that with ocrelizumab.

### 1.4. Ofatumumab

Ofatumumab is currently being evaluated in phase 3 clinical trials for the treatment of relapsing MS (Table 1). Ofatumumab is a fully humanized MAB, which binds to the human CD20 antigen inducing B-cell lysis through ADCC and CDC. Its target epitope is located in a different cellular site than rituximab and ocrelizumab [49,50].

A small phase II study was completed which showed a reduction in new MRI lesions of 99% for all dose groups versus placebo by 24 weeks [50]. The MIRROR study, which compared ofatumumab to placebo in a phase IIB trial using subcutaneous dosing, showed a reduction in cumulative new gadolinium lesions of 65% for all dose groups when compared to placebo (*p* < 0.001). For all doses of >30 mg a reduction of >90% new brain lesions was seen over a 12 week period. The most common adverse event was injection related reactions at the first dose, which decreased in subsequent doses [49]. 

Observed adverse events included rash and urticaria, infusion reactions, pruritus, headaches, nasopharyngitis, hypersensitivity and dyspnea [50,51]. None of the patients developed human anti-human antibodies. Marginal changes in IgG, IgA, and IgM were observed [50]. Monitoring is similar to that with ocrelizumab.

### 1.5. Ublituximab

Ublituximab is a novel glycoengineered chimeric MAB antiCD20 therapy that is currently in phase III studies (ULTIMATE) (Table 1). Glycoenginering allows it to have enhanced affinity for Fc γ RIIIa receptors, eliciting increased ADCC. At week 4, a 99% B cell depletion was observed and maintained up to week 24 after treatment [52]. One particular benefit is that the infusion time duration is typically between 1 and 2 h.

Results from a phase II study with a median duration of 11 months, showed that ublituximab at week 24 has an ARR of 0.05 and a reduction in new T2 lesion volume of 7.67% compared to baseline. Additionally at week 24, 98% of participants were relapse free, no subjects had a contrast enhancing lesion, 84% did not have any new/enlarging T2 lesion, and 93% did not have confirmed disability progression at 24 weeks resulting in 76% of subjects meeting criteria for no evidence of disease activity [52]. 

No grade 3–4 AEs were observed, all IRRs reported were levels 1 and 2. Some adverse reactions reported include mild/moderate IRRs, fatigue, headache, numbness, common colds, dizziness, nausea/vomiting, and upper respiratory infection [52]. Monitoring is similar to that with ocrelizumab.

### 1.6. Alemtuzumab (Lemtrada™)

Alemtuzumab is a humanized anti-CD52 cytolytic MAB which targets the surface of lymphocytes and monocytes predominantly by ADCC. The repopulation of B cells occurs within 6 weeks of treatment, but T cells take longer to normalize at 9–12 months after a course of treatment [53,54]. This provides a long pharmacodynamic effect allowing the medication to be administered intravenously over two treatment courses. The first treatment consist of 12 mg/day on 5 consecutive days with the second on 3 consecutive days administered 12 months after the first treatment [53,54].

As a first line treatment, alemtuzumab in CARE-MS 1 showed an ARR of 54%, a non-significant reduction in sustained accumulation of disability, a 17% reduction in new or enlarging T2 lesion, a 63% reduction in contrast enhancing lesions, and a 42% reduction in brain parenchymal atrophy compared with IFN-β1a [53]. In CARE-MS 2, alemtuzumab showed an ARR of 50%, a non-significant reduction in sustained accumulation of disability, a 32% reduction in new or enlarging T2 lesion, a 61% reduction in contrast enhancing lesions, and a non-significant reduction in brain parenchymal atrophy compared with IFN-β1a [54]. Over 5 years, it lowered the risk of sustained accumulation of disability by 72% compared with IFN-β1a (*p* < 0.0001) and reduced the number of relapses by 69% over IFN-β1a (*p* < 0.0001) [54]. Both studies were performed in a non-blinded fashion, meaning patients knew if they were under the experimental arm of the trial and being prescribed this MAB. This approach was adopted due to the severity of the IRRs, nonetheless, blinded neurologists performed the efficacy assessments [55].

Adverse events have created the need for a REMS program. For example, recently the FDA issued a warning about elevated risk of stroke and artery damage, for this reason a black box label was added to the U.S. product label [56]. Patients can develop other autoimmune conditions such as thyroid disease (40.7%) [57], as well as immune thrombocytopenia, autoimmune hemolytic anemia, and anti-glomerular basement membrane disease [53,54]. Additionally, there has been an increased risk of malignancies such as thyroid cancer, melanoma, and lymphoproliferative disorders. IRRs are also common (>90%) [53,58], including rare cases of serum sickness and anaphylaxis. Over time, infections (>71%) can also occur including nasopharyngitis, urinary tract infections, upper respiratory tract infections, herpes viral infection, and fungal infections [59]. The monitoring labs that constitute the REMS program include complete blood count with differential, serum creatinine, urinalysis with microscopy, which should be performed monthly, and thyroid function tests, which should be done every 3 months, until 48 months after the last infusion [60]. 

### 1.7. Opicinumab

It is encouraging to see new MABs being developed for repair/remyelination of lesions in MS patients. Opicinumab is a fully humanized MAB that targets LINGO-1. By inhibiting LINGO-1, oligodendrocyte precursor cells (OPCs) can differentiate into mature oligodendrocytes (OLCs) and allow for remyelination of damaged plaques (Table 1, Figure 2) [9,38,61]. 

Opicinumab was evaluated in a phase II study (RENEW) as an add-on in patients with optic neuritis where it did not differ significantly in remyelination rates when compared to placebo at week 24. However, there was a small improvement of 9.1 milliseconds on full field visual evoked responses in the per protocol analysis that was not significant in the intention to treat analysis [9]. Subgroup analysis showed an improvement for baseline age ≥33 years of −14.17 milliseconds suggesting a greater benefit in older patients [62].

Another phase II study (SYNERGY) evaluated the efficacy of coadministration of opicinumab with IFN-β1a. The primary end point was the percentage of patients with an improvement over at least 3 months on a multicomponent endpoint comprising the expanded disability status scale, timed 25 foot walk, 9-hole peg test, and a 3-s paced auditory serial addition test. The percentage of improvement was 51.6% for placebo, 51.1% for 3 mg/kg opicinumab, 65.6% for 10 mg/kg, 68.8% for 30 mg/kg, and 41.2% for 100 mg/kg. In summary, there was an increased percentage of remyelination with opicinumab at 10 and 30 mg/kg when compared to placebo [63]. An additional study is underway (AFFINITY), which looks at patients that did better in SYNERGY, and includes those with disease activity of less than 21 years and that meet certain criteria on magnetization transfer ratio and diffusion tensor imaging on magnetic resonance scans. 

Opicinumab has been well tolerated, adverse events have been similarly reported among groups treated with opicinumab and placebo [62,63]. However, some mild hypersensitivity reactions have been reported [61].

## 2. New Horizons and Future Trends for Therapeutic Monoclonal Antibodies

MABs have become a mainstay of MS treatment and they are likely to continue to be developed and optimized for the treatment of this disease, such as analyzing the safety of faster infusion times and administration by subcutaneous routes. MABs are some of the most effective therapies for relapsing MS without the side effects associated with chemotherapeutic agents. Additionally, new MABS are being developed to help repair the damage/disability that has already occurred, which is promising. New advancements in bioengineering are being incorporated to antibodies that are more humanized to help optimize their effectiveness and improve their tolerability and safety. The newer MABs that are being developed (Table 1) are based on lessons learned from past studies and drug development programs of drugs that have already been approved (Table 1) or stopped (Table 2). Overall, it is expected that MABs will continue to be a preferred therapy for MS in the foreseeable future.

## Figures and Tables

**Table 1:** Monoclonal antibodies currently on the market or being evaluated in phase II/III studies.

	Therapeutic Monoclonal Antibodies				
MAB	Composition	Target	Mechanism of Action	Administration	FDA Approval Date for MS
Alemtuzumab	Humanized MAB IgGk	CD52	ADCC	Intravenous	November 2014
Elezanumab [11]	Fully human MAB	RGMa	Binds and neutralizes RGMa which modulates T cell responses and dendritic cells in CNS lesions	Intravenous	N/A
GNbAC1 [12]	Humanized IgG4 MAB	Envelope protein of HERV-W MSRV	Targets the envelope protein of HERV-W MSRV, which may play a critical role in multiple sclerosis	Intravenous	N/A
Natalizumab	Humanized monoclonal IgG1	Cell adhesion molecule α4-integrin	Preventing lymphocyte transport across the blood brain barrier	Intravenous	November 2004 and reapproved on June 2006
Ocrelizumab	Humanized IgG1	Phosphorylated glycoprotein CD20 on B lymphocytes	ADCC > CDC	Intravenous	March 2017
Ofatumumab	Fully humanized IgG1	CD20	CDC > ADCC	Subcutaneous	N/A
Opicinumab	Humanized MAB	Targets LINGO-1	Allows OPCs to differentiate into mature OLG for remyelination	Intravenous	N/A
Ublituximab	Chimeric IgG1 MAB	CD20	CDC and ADCC	Intravenous	N/A
Rituximab	Chimeric (murine/human) MAB	CD20	CDC and ADCC	Intravenous	N/A
VAY736 [13]	Defucosylated, human IgG1 MAB	Targets the receptor for BAFF-R	ADCC and blockade of BAFF:BAFF-R signaling that drives B cell differentiation, proliferation and survival	Intravenous	N/A

Abbreviations: monoclonal antibody (MAB), multiple sclerosis (MS), not applicable (N/A), envelope protein (Env), human endogenous multiple sclerosis-associated retrovirus (HERV-W MSRV), cluster of differentiation (CD), complement-dependent cytotoxicity (CDC), antibody-dependent cellular cytotoxicity (ADCC), T helper (Th), Leucine-rich repeat and immunoglobulin domain-containing neurite outgrowth inhibitor receptor-interacting protein 1 (LINGO-1), oligodendrocyte precursor cells (OPCs), oligodendrocytes (OLGs), repulsive guidance molecule A (RGMa), B cell activating factor of the TNF family (BAFF-R).

**Table 2:** Examples of MAB therapies that have been discontinued from further clinical trials due to lack of efficacy or serious adverse events in the treatment of multiple sclerosis.

MAB	Composition	Target/Mechanism	Withdrawn
Atacicept [14]	Fully humanized recombinant fusion protein containing the extracellular ligand-binding portion of the human TACI receptor	Binds to the cytokines BLyS and APRIL, involved in B-cell differentiation, maturation, and survival.	Increases relapse rates in multiple sclerosis reflected on an increase in annualized relapse rates.
Daclizumab [15]	Humanized IgG1 MAB	CD25, which is attached to the Tac epitope on the alpha chain of CD25 (IL-2 receptor) on activated lymphocytes	Post-marketing vigilance helped to detect secondary autoimmune events, including inflammatory encephalitis in 12 patients worldwide leading to at least 3 deaths where an interaction with the drug could not be ruled out.
Muromonab [16]	Chimeric MAB, first MAB to ever be approved	Inhibition of CD3 receptor	High toxicity made it unlikely to be a preferred treatment for MS.
Secukinumab [17]	Humanized IgG1kappa MAB	IL-17 receptor, inhibits proinflammatory IL-17A	Discontinued due to the development of a fully-human anti IL-17 MAB with better potential
Tabalumab [18]	Selective and fully human IgG4 MAB	Neutralization of membrane-bound and soluble B-cell activating factor (BAFF)	Results from phase 2 clinical trials in patients with RMS, showed no evidence of reduction Gd-enhancing lesions versus placebo, further analysis were discontinued.
Ustekinumab [19]	Fully humanized IgG1 MAB	Targets subunit P40 on cytokines IL-12 and IL-23 preventing them from differentiating and activating Th1 cells	Discontinued after phase 2 trials for low/lack of efficacy.
Vatelizumab [20]	Fully humanized MAB that targets VLA-2, a collagen binding integrin expressed on activated lymphocytes	Preventing the crossing of inflammatory cells into the brain, reducing inflammation and tested on RMS	Primary efficacy endpoint was not met after phase 2a and 2b studies halting further development for MS.

Abbreviations: transmembrane activator and calcium modulator and cyclophilin-ligand interactor (TACI), B-lymphocyte stimulator, also known as TNFSF20 (BLyS), a proliferation-inducing ligand, also known as TNFSF13 (APRIL), monoclonal antibody (MAB), T activation (Tac), cluster of differentiation (CD), interleukin (IL-), B-cell activating factor (BAFF), relapsing multiple sclerosis (RMS), gadolinium (Gd), T helper (Th), very late antigen-2 (VLA-2; also known as integrin α2β1).
